# O.3.1-4 Implementation of the JOGG approach (Healthy Youth, Healthy Future): unpacking the complex implementation process of community-based health promotion using the Critical Event Card

**DOI:** 10.1093/eurpub/ckad133.141

**Published:** 2023-09-11

**Authors:** Irma Huiberts, Amika Singh, Dorine Collard, Mara Hendriks, Frank van Lenthe, Mai Chinapaw

**Affiliations:** Department of Public and Occupational Health, Amsterdam UMC location Vrije Universiteit Amsterdam, Amsterdam, The Netherlands; Mulier Instituut, Utrecht, The Netherlands; Mulier Instituut, Utrecht, The Netherlands; Mulier Instituut, Utrecht, The Netherlands; Mulier Instituut, Utrecht, The Netherlands; Department of Public Health, Erasmus Medical Centre, Rotterdam, The Netherlands; i.huiberts@amsterdamumc.nl; Department of Public and Occupational Health, Amsterdam UMC location Vrije Universiteit Amsterdam, Amsterdam, The Netherlands

## Abstract

**Purpose:**

community-based programmes are important for population health promotion. Due to the context dependent and dynamic nature of these programmes, it is challenging to define and unravel successful implementation. Therefore, few studies have examined how successful implementation can be accomplished. We studied the implementation of the Dutch JOGG (Healthy Youth, Healthy Future) approach, a community-based programme for promoting physical activity and healthy nutrition in youth. The aim of our study was to (1) understand how the implementation a community-based programme changes over time, in interaction with the local context, and (2) identify key processes that influence implementation.

**Methods:**

to guide data collection and analysis we applied the Critical Event Card (CEC). This analytical tool conceptualizes the implementation process as a continuous and dynamic interaction between the programme (actors) and context. The CEC aims to identify critical events in the evolution of the programme. We collected programme documents and conducted 24 (group) interviews about the implementation of the JOGG approach in 9 different communities. We analysed the data according to the CEC.

**Results:**

over 6-10 years of implementation, JOGG communities included in the current study had changed their implementation strategy, integrated JOGG with other policy initiatives, scaled up to new neighbourhoods and later regained focus to stimulate health equity. Critical events that elicited these changes included (1) how JOGG was framed in local policy during the adoption process, (2) new national health policies, (3) increased national attention for health equity, (4) scale-up, (5) staff turnover and (6) the implementation teams’ experiences (successes or failures). An opportunity for change (e.g. new policy term) was needed to trigger formal programme changes. How critical events influenced implementation depended on the context and implementation team.

**Conclusions:**

when studying the implementation of community-based programmes it is important to take into account their dynamic and context dependent nature. By looking at critical events in the interaction between programme and context over time, we were able to identify key processes in implementation. These findings can support future implementation of community-based programmes.

**Funding:**

by the national JOGG organisation, supported by the Dutch ministry of Public Health, Welfare and Sport.

